# Case report: first symptomatic *Candidatus* Neoehrlichia mikurensis infection in Slovenia

**DOI:** 10.1186/s12879-021-06297-z

**Published:** 2021-06-15

**Authors:** Mitja Lenart, Miha Simoniti, Katja Strašek-Smrdel, Vesna Cvitković Špik, Tanja Selič-Kurinčič, Tatjana Avšič-Županc

**Affiliations:** 1 Department of Infectious diseases, General Hospital Celje, Oblakova ulica 5, 3000 Celje, Slovenia; 2 Faculty of Medicine, Institute of Microbiology and Immunology, University of Ljubljana, Zaloška cesta 4, Ljubljana, 1000 Slovenia

**Keywords:** Tick, *Candidatus* Neoehrlichia mikurensis, Infection, Fever of unknown origin, 16S rRNA PCR, Immunocompromised

## Abstract

**Background:**

*Candidatus* Neoehrlichia mikurensis (CNM) is an emerging tick-born pathogen and usually causes symptomatic infection only in immunocompromised patients. Apart from one described case found in the literature where cultivation was successful, all cases so far were diagnosed by using broad-range 16S rDNA PCR.

**Case presentation:**

Our patient presented with a prolonged febrile state of unknown origin. Clinical presentation, extensive medical workup and classic microbiologic testing were non-conclusive. Several infectious agents and other causes for the febrile state were excluded. In the end, a broad-range 16S rDNA PCR was to be performed to confirm the diagnosis of CNM infection. Treatment was successful with doxycycline.

**Conclusions:**

Due to the obscurity of the pathogen, diagnostic workup in CNM is prolonged and challenging. More awareness is need about this emerging infectious disease in countries with high prevalence of tick-borne diseases as standard microbiological methods are not successful in confirming the diagnosis.

**Supplementary Information:**

The online version contains supplementary material available at 10.1186/s12879-021-06297-z.

## Background

In 2010, the first case of an immunocompromised patient with recurrent episodes of fever was described in Sweden [1]. After prolonged diagnostic workup and antibiotic treatment, the fever persisted. Amplification of 16S rRNA gene sequences from blood and sequence analysis revealed “*Candidatus* Neoehrlichia mikurensis”, a tick-borne pathogen never before described in humans [2]. The first successful cultivation and propagation of CNM from clinical isolates was described in 2019 [3]. The most common signs and symptoms of symptomatic CNM infection are recurrent fever, headache, malaise and myalgia. Fewer patients experience arthralgia, cough, nausea, vomiting, diarrhoea and a nonspecific rash. Laboratory findings are similar to those found in acute anaplasmosis and ehrlichiosis: elevated CRP, elevated hepatic enzymes, anaemia, thrombocytopenia, leukopenia or leucocytosis [1, 2].

## Case presentation

### Clinical presentation, workup timeline and treatment

A 66-year-old female patient presented to the Emergency department (Nov.4th 2019) with a recurrent fever lasting for 14 days. The fever occurred daily, was high grade (39 °C/102 °F), usually occurred in evenings and ceased after taking paracetamol. No night sweats were present. She had mild dyspnoea on exertion and worsening of otherwise chronic cervicalgia. Her medical history revealed she recently (Oct.10th 2019) received her 8th cycle of chemotherapy with R-CHOP (rituximab, cyclophosphamide, doxorubicin, vincristine, prednisone) due to a disseminated diffuse large B-cell lymphoma. She had a medical history of resection of a malignant rectal tumour with reported full remission (from 2011), splenectomy, and chronic gastritis, presence of a mechanical artificial aortic valve on anticoagulation and chronic anaemia. She was treated with amoxicillin/clavulanic acid (1000 mg/12 h p.o.) 3 months previously for 10 days due to fever of unknown origin (FUO) with concomitant thrombocytopenia (41.000/mcL) and anaemia (Hb 8.2 g/dL). Epidemiologic history was unremarkable in the last 6 months: she denied travel, contact with animals, tick or insect bites, or eating raw foods.

Physical examination revealed no abnormalities apart from a fever of 39 °C and the closing click of the mechanical aortic valve without obvious murmurs.

The chest X-ray was normal with a visible ring of the artificial valve. In laboratory studies, a moderate normocytic anaemia (9.4 g/dL) and hyponatremia (133 mmol/L) were present. CRP was elevated (111 mg/L), leukocytes and thrombocytes were normal, as were the estimated erythrocyte sedimentation rate, renal function and aminotransferases.

An infectious disease specialist was consulted, blood cultures were drawn, empirical antibiotic treatment with amoxicillin/clavulanic acid (1200 mg/8 h i.v.) was started and the patient was admitted to the hospital for further workup (Nov.4th 2019). During the next 3 days, the patient remained febrile; TSH, LDH, HIV serology, and blood cultures were normal or negative and an abdominal ultrasound showed a small regenerate of the spleen. After consulting with an infectious disease specialist again, antibiotic treatment was escalated to flucloxacillin (2 g/6 h i.v.) and ceftriaxone (2 g/24 h i.v.). Results of further microbiological workup (urinary Legionella antigen, respiratory PCR panel, blood cultures, urine culture, stool PCR panel, 1,3-beta-D-glucan, serologic testing for *Coxiella burnetii*, *Brucella* sp., cytomegalovirus, Epstein–Barr virus, leptospirosis, Hantaviruses), were all negative. As anaemia worsened, the patient received several transfusions of concentrated erythrocytes. A transthoracic and transoesophageal echocardiography (TTE and TEE) showed no signs of infectious endocarditis. Fever persisted and after 3 days, antibiotic therapy was again escalated to vancomycin (1 g/12 h i.v.) and cefepime (2 g/8 h i.v.). An esophagogastroduodenoscopy showed chronic gastritis with no signs of recent gastric bleeding. A slight redness of the left breast was observed but a gynaecological consultation excluded mastitis. As the cause of fever remained unknown despite an extensive work-up, a PET CT scan was performed and showed non-specific activity around the artificial heart valve. After 14 days of therapy with vancomycin and cefepime, the inflammatory markers were significantly lower but still elevated (CRP of 36 mg/L), anaemia was stable (9,3 g/dL) and thrombocyte count was normal (221.000/mcL). The patient gradually became sub to afebrile (body temperature remained below 38 °C). As there were no signs of bacterial infection antibiotic treatment was discontinued and the patient’s oncologist was consulted. It was concluded that there were no signs of lymphoma progression but further follow-up was needed. The patient was discharged from hospital on November 21th 2019.

During the subsequent 2 months, the patient still had occasional episodes of fever, chills, malaise and started having night sweats. A control TEE was performed where endocarditis was again excluded. An infectious disease specialist was consulted again; 1,3-beta-D-glucan and serology for *C. burnetii* and *Brucella* sp*.* were repeated and whole blood was sent for bacterial 16S rDNA PCR (29th January 2020).

While serology remained negative, the 16S rDNA PCR and additional specific PCR for CNM from the blood sample were both positive for CNM. Treatment with doxycycline 100 mg/12 h p.o. was started.

The patient rapidly defervesced after starting treatment with doxycycline and her symptoms completely subsided. On follow up, after 12 days of treatment with doxycycline, the patient was seen at a control appointment. A whole blood and serum sample were sent to the Institute of Microbiology and Immunology for CNM specific PCR detection and detection of antibodies against *Anaplasma phagocytophilum*, respectively; PCR was still positive for CNM, while detection of antibodies against *A. phagocytophilum* was negative. She also developed neutropenia. Treatment with doxycycline was prolonged until February 28th 2020 for 4 weeks in total and was discontinued after two subsequent specific PCRs were negative (on February 20th and February 27th 2020). At her latest follow up, no neutropenia was present, no new symptoms had appeared and the patient started seeing her oncologist again. An Indium-111 white blood cell scintigraphy was performed on early follow up, which excluded a septic or infectious process in the left atrium and ventricle, though some nonspecific activity was observed. On long-term follow up for the following 12 months, the patient remained afebrile and successfully finished her chemotherapy.

### Microbiological analyses

A broad-range 16S rDNA PCR was performed on the patient’s blood sample. Patient’s whole blood with EDTA anticoagulant was centrifuged for 10 min at 800 rpm. A plasma sample (1.5 mL) was collected and concentrated at 14500 rpm for 15 min. Four hundred μL of a lower portion of the centrifuged plasma sample including pellet was used for nucleic acid extraction with MagNA Pure 24 Total NA Isolation Kit (Roche, Germany) according to the Pathogen 2.0 purification protocol with the input sample volume of 200 μL and the elution volume of 100 μL. A broad-range 16S rDNA PCR [4] with subsequent sequencing revealed CNM in the patient’s blood sample. An identity match of 97.6% was revealed, using Sequence Match online RDP tool [5]. A positive control for a broad-range 16S rDNA PCR was *Staphylococcus aureus* from a pure culture, a negative control was molecular biology grade water. For the confirmation of a detected bacterial pathogen, additional conventional PCRs for a part of *groEL* gene [6] and for a larger part of 16S rDNA [7] were performed, both with subsequent sequencing. The sequences were assembled by CLC Main Workbench, version 6.1 software (CLC bio, Aarhus, Denmark) and analyzed with MEGA 5.05 software [8]. The results after analysis of sequences matched 100% to sequences of CNM from human samples from Germany (Acc. No. EU810406 and Acc. No. EU810404, respectively). Phylogenetic analysis grouped Slovenian patient’s sequence of CNM in European cluster of *groEL* and 16S rDNA gene sequences from CNM (Additional file Figure 1, Additional file-Figure 2). Positive control for confirmatory PCRs was CNM extracted from a small mammal in Slovenia negative control was molecular biology grade water (Filling the gap on the knowledge of Candidatus Neoehrlichia mikurensis in Europe, Slovenia. In: ESCC AR - ASR Joint Meeting. Marseille; 2017 "unpublished data").

For the follow up and for control of treatment, additional whole blood samples with EDTA anticoagulant were obtained. Total nucleic acid extraction from 400 μL plasma sample was done by EZ1 Virus Mini Kit v2.0 according to the manufacturer’s instructions (Qiagen, Germany). Eluates were tested by a specific real-time PCR for *groEL* gene [9]. Positive and negative controls were the same as for the confirmatory PCRs. The patient’s samples obtained 12 days later were still positive, but those obtained three and 4 weeks later at additional control visits were negative.

Additionally, an immunofluorescence assay (IFA) for detection of antibodies was performed in sera sent on initial date and 2 weeks later. Antigens used were of *A. phagocytophilum* (in-house assay) and of *Ehrlichia chaffeensis* (Focus Diagnostics, USA), as IFA for detection of antibodies against CNM is not yet available. Antibodies against *A. phagocytophilum* or *E. chaffeensis* antigens were not detected.

## Discussion

This case report describes the first ever-confirmed human infection with CNM in Slovenia, where the prevalence of other tick-borne infections (i.e. borreliosis) is relatively high compared to other European countries [10].

As in some other European countries, the presence of CNM in ticks and field mice in Slovenia has been known for some time (unpublished data), which seem to be the main reservoir for the pathogen.

Similar to other described cases, this patient with symptomatic CNM infection was immunocompromised and the diagnostic workup of FUO was prolonged [11, 12]. This was due to a lack of specific biochemical or clinical clues to guide the physicians and the obscurity of the pathogen itself. The patient did not notice a tick bite; it is estimated, however, that about a third of people do not notice a tick bite, but develop a tick-borne disease [13]. As in other case-reports, the diagnosis of CNM infection was made using molecular methods. Our patient had a previous episode of a febrile illness with no known cause 3 months before hospitalization, but clinically improved with amoxicillin/clavulanic acid. We assume that was a different condition.

There are no specific recommendations on choice of antibiotic; however, as with most bacteria from the family Anaplasmataceae, doxycycline 100 mg/12 h p.o. for 3–4 weeks is the most often prescribed option with good clinical outcome. Rifampicin seems to be a reasonable alternative [2].

No clear reason for the transient neutropenia in the described case was found and the patient did not develop opportunistic infections. It could have been caused by doxycycline as it is a reported side effect per the summary of product characteristics.

## Conclusions

*Candidatus* Neoehrlichia mikurensis seems to be an emerging tick-borne pathogen mostly presenting as a prolonged intermittent episodes of fever in immunocompromised patients. As there are no specific biochemical markers, clinical signs or serological assays and the cultivation from blood cultures is unsuccessful, the only method of confirming the diagnosis so far is the specific and/or broad range 16rRNA PCR. Four weeks of doxycycline was effective for the case described above.

## Supplementary Information

**Additional file 1: Table 1.** Primers and their sequences for PCRs and sequencing.

**Additional file 2: Figure 1.** Phylogenetic tree of operon *groEL* sequences inferred using Maximum Likelihood method based on the Tamura-Nei model (1). The scale barr indicates the number of base substitutions per site (1766 positions).

**Additional file 3: Figure 2.** Phylogenetic tree of 16S rDNA gene sequences inferred using Maximum Likelihood method based on the Tamura-Nei model (1). The scale barr indicates the number of base substitutions per site (1516 positions).

## Data Availability

Data sharing is not applicable to this article as no datasets were generated or analysed during the current study.

## Abbreviations

CNM: *Candidatus* Neoehrlichia mikurensis
R-CHOP: Cyclophosphamide, doxorubicin, vincristine, and prednisone
CRP: C-reactive protein
TSH: Thyroid stimulating hormone
LDH: Lactate dehydrogenase
HIV: Human immunodeficiency virus
PET/CT: Positron emission tomography–computed tomography
TEE: Transoesophageal echocardiography
TTE: Transthoracic echocardiography
IFA: Immunofluorescence assay
PCR: Polymerase chain reaction
FUO: Fever of unknown origin
