# Supplementary material for: Case report: first symptomatic Candidatus Neoehrlichia mikurensis infection in Slovenia
Source: BMC Infect Dis. 2021 Jun 15;21:579. doi: 10.1186/s12879-021-06297-z (PMC8207769; doi:10.1186/s12879-021-06297-z)
Supplement: Supplementary file 1 — Additional file 1: Table 1. Primers and their sequences for PCRs and sequencing. [file 12879_2021_6297_MOESM1_ESM.docx]

Table 1: Primers and their sequences for PCRs and sequencing

| Primer name | sequence of the primer | target gene | used for: | reference |
| --- | --- | --- | --- | --- |
| 16S FA | 5'-GCTCAGATTGAACGCTGG-3' | 16S rDNA | routine PCR | 1 |
| 16S FB | 5'-GCTCAGGAYGAACGCTGG-3' |  |  |  |
| 16S SR | 5'-TACTGCTGCCTCCCGTA-3' |  |  |  |
| NMikGroEL-F2 | 5'-CCTTGAAAATATAGCAAGATCAGGTAG-3' | groEL | specific real-timePCR | 2 |
| NMikGroEL-rev1 | 5'-CCACCACGTAACTTATTTAGCACTAAAG-3' |  |  |  |
| NMikGroEL-rev2 | 5'-CCACCACGTAACTTATTTAGTACTAAAG-3' |  |  |  |
| NMikGroEL-P2a | 6FAM-CCTCTACTAATTATTGCTGAAGATGTAGAAGGTGAAGC-BHQ1 |  |  |  |
| 1512r | 5'-ACG GCT ACC TTG TTA CGA CTT C-3' | 16S rDNA | conventional PCR | 3 |
| 8f | 5'-AGA GTT TGA TCM TGG CTC AG-3' |  |  |  |
| Nehr16Sre | 5'-AGC CAA ACT GAC TCT TCC G-3' |  | nested PCR and sequencing |  |
| 8f | 5'-AGA GTT TGA TCM TGG CTC AG-3' |  |  |  |
| Nehr16Sfo | 5'-GCG ACT ATC TGG CTC AGT T-3' |  |  |  |
| 1512r | 5'-ACG GCT ACC TTG TTA CGA CTT C-3' |  |  |  |
| NM-128s | 5'-AACAGGTGAAACACTAGATAAGTCCAT-3' | groEL | conventional PCR and sequencing | 4 |
| NM-1152as | 5'-TTCTACTTTGAACATTTGAAGAATTACTAT-3' |  |  |  |

Reference:

1. Harris KA, Hartley JC. Development of broad-range 16S rDNA PCR for use in the routine diagnostic clinical microbiology service. J Med Microbiol. 2003 Aug;52(Pt 8):685-691.
2. Jahfari S, Fonville M, Hengeveld P, Reusken C, Scholte E-J, Takken W, et al. Prevalence of Neoehrlichia mikurensis in ticks and rodents from North-west Europe. Parasit Vectors. 2012 Dec 19;5(74).
3. von Loewenich FD, Geissdorfer W, Disque C, Matten J, Schett G, Sakka SG, et al. Detection of ‘*Candidatus* Neoehrlichia mikurensis’ in Two Patients with Severe Febrile Illnesses: Evidence for a European Sequence Variant. J Clin Microbiol. 2010 Jul 1;48(7):2630–5.
4. Diniz PPVP, Schulz BS, Hartmann K, Breitschwerdt EB. ‘*Candidatus* Neoehrlichia mikurensis’ Infection in a Dog from Germany. J Clin Microbiol. 2011 May 1;49(5):2059–62.
