# Supplementary material for: Case report: first symptomatic Candidatus Neoehrlichia mikurensis infection in Slovenia
Source: BMC Infect Dis. 2021 Jun 15;21:579. doi: 10.1186/s12879-021-06297-z (PMC8207769; doi:10.1186/s12879-021-06297-z)
Supplement: Supplementary file 2 — Additional file 2: Figure 1. Phylogenetic tree of operon groEL sequences inferred using Maximum Likelihood method based on the Tamura-Nei model (1). The scale barr indicates the number of base substitutions per site (1766 positions). [file 12879_2021_6297_MOESM2_ESM.docx]

*I. ricinus* Sweden KF849343

*I. ricinus* Poland KF312363

Human Germany EU810406

*I. ricinus* Germany EU810407

Human patient SLO

Dog Germany EU432375

Human China JQ359062

*H. concinna* China JQ359078

Rat Japan AB084583

*E. chaffeensis* NC 007799

*A. phagocytophilum* NC 021881

0.05

Figure 1: Phylogenetic tree of operon *groEL* sequences inferred using Maximum Likelihood method based on the Tamura-Nei model (1). The scale barr indicates the number of base substitutions per site (1766 positions).

Tamura K. and Nei M. (**1993**). Estimation of the number of nucleotide substitutions in the control region of mitochondrial DNA in humans and chimpanzees. *Molecular Biology and Evolution* **10**:512-526.
