# Supplementary material for: Case report: first symptomatic Candidatus Neoehrlichia mikurensis infection in Slovenia
Source: BMC Infect Dis. 2021 Jun 15;21:579. doi: 10.1186/s12879-021-06297-z (PMC8207769; doi:10.1186/s12879-021-06297-z)
Supplement: Supplementary file 3 — Additional file 3: Figure 2. Phylogenetic tree of 16S rDNA gene sequences inferred using Maximum Likelihood method based on the Tamura-Nei model (1). The scale barr indicates the number of base substitutions per site (1516 positions). [file 12879_2021_6297_MOESM3_ESM.docx]

Human Switzerland GQ501090

*I. ricinus* Netherlands AF104680

Human Germany EU810404

Human patient SLO

*I. ricinus* Germany EU810405

Human China JQ359045

*A.peninsulae* Russia FJ966366

*E. chaffeensis* NC 007799

*A. phagocytophilum* NC 021881

0,0100

Figure 2: Phylogenetic tree of 16S rDNA gene sequences inferred using Maximum Likelihood method based on the Tamura-Nei model (1). The scale barr indicates the number of base substitutions per site (1516 positions).

1. Tamura K. and Nei M. (**1993**). Estimation of the number of nucleotide substitutions in the control region of mitochondrial DNA in humans and chimpanzees. *Molecular Biology and Evolution* **10**:512-526.
